# Physicochemical and Biological Properties of Quercetin-Loaded Low-Molecular-Weight Chitosan Nanoparticles Derived from *Hermetia illucens* Larvae and Crustacean Sources: A Comparative Study

**DOI:** 10.3390/pharmaceutics17081016

**Published:** 2025-08-05

**Authors:** Anna Guarnieri, Rosanna Mallamaci, Giuseppe Trapani, Dolores Ianniciello, Carmen Scieuzo, Francesco Iannielli, Luigi Capasso, Maria Chiara Sportelli, Alessandra Barbanente, Michela Marsico, Angela De Bonis, Stefano Castellani, Patrizia Falabella, Adriana Trapani

**Affiliations:** 1 Department of Basic and Applied Sciences, University of Basilicata, 85100 Potenza, Italy; anna.guarnieri@unibas.it (A.G.); dolores.ianniciello@unibas.it (D.I.); carmen.scieuzo@unibas.it (C.S.); francesco.iannielli003@unibas.it (F.I.); michela.marsico@unibas.it (M.M.); angela.debonis@unibas.it (A.D.B.); 2 Department of Biosciences, Biotechnologies and Environment, University of Bari “Aldo Moro”, 70125 Bari, Italy; rosanna.mallamaci@uniba.it; 3 Department of Pharmacy-Drug Sciences, University of Bari “Aldo Moro”, 70125 Bari, Italy; giuseppe.trapani@uniba.it (G.T.); l.capasso2@phd.uniba.it (L.C.); 4 Spinoff XFlies S.R.L, University of Basilicata, 85100 Potenza, Italy; 5 Chemistry Department, University of Bari “Aldo Moro”, 70125 Bari, Italy; maria.sportelli@uniba.it (M.C.S.); alessandra.barbanente@uniba.it (A.B.); 6 Department of Precision and Regenerative Medicine and Ionian Area (DiMePRe-J), University of Bari “Aldo Moro”, 70125 Bari, Italy; stefano.castellani@uniba.it

**Keywords:** insect source, chitosan nanoparticles, quercetin, X-ray diffraction, antioxidant effect, antimicrobial activity

## Abstract

**Introduction**. Larvae of the insect *Hermetia illucens* can represent an alternative source for low-molecular-weight chitosan (CS) production compared with CS from crustaceans (CS_crustac_), making it appealing in terms of pharmaceutical applications. Hence, the performances of CS_larvae_ and CS_crustac_ were compared herein by investigating the in vitro features of nanoparticles (NPs) made from each polysaccharide and administered with the antioxidant quercetin (QUE). **Methods**. X-ray diffraction and FT-IR spectroscopy enabled the identification of each type of CS. Following the ionic gelation technique and using sulfobutylether-β-cyclodextrin as a cross-linking agent, NPs were easily obtained. **Results.** Physicochemical data, release studies in PBS, and the evaluation of antioxidant effects via the 1,1-diphenyl-2-picrylhydrazyl (DPPH) test were studied for both CS_larvae_ and CS_crustac_. QUE-loaded NP sizes ranged from 180 to 547 nm, and zeta potential values were between +7.5 and +39.3 mV. In vitro QUE release in PBS was faster from QUE-CS_larvae_ NPs than from CS_crustac_, and high antioxidant activity—according to the DPPH test—was observed for all tested NP formulations. **Discussion.** The agar diffusion assay, referring to *Escherichia coli* and *Micrococcus flavus,* as well as the microdilution assay, showed the best performance as antimicrobial formulations in the case of QUE-CS_larvae_ NPs. QUE-CS_larvae_ NPs can represent a promising vehicle for QUE, releasing it in a sustained manner, and, relevantly, the synergism noticed between QUE and CS_larvae_ resulted in a final antimicrobial product. **Conclusions.** New perspectives for low-molecular-weight CS are disclosed by adopting renewable sources from insects instead of the commercial CS_crustac._

## 1. Introduction

Chitosan (CS), a linear polysaccharide consisting of β-1,4-D-glucosamine and N-acetyl-D-glucosamine units, is globally recognized as one of the most important polymers obtained from natural and renewable sources with many relevant applications in pharmaceutical, cosmetic, and food industries, among others [1,2].

Obviously, many of the CS applications are strictly related to their peculiar structures and features. Indeed, like most polysaccharides, CS is a non-toxic, biodegradable, and biocompatible polymer with bioadhesive and immunomodulatory properties [3,4]. Thereby, CS is a polycation capable of providing efficient electrostatic interactions between its positively charged amino groups and negatively charged carboxylic and sulphate moieties of the polyanionic mucin chains of the mucosal layer [5]. This is why it is most employed in the pharmaceutical field, i.e., to prepare dosage forms that are useful for transmucosal delivery. Moreover, due to its mucoadhesive properties, CS allows for a greater retention of these forms on the mucosa, which can lead to improved drug bioavailability at the site of action [5].

Similarly, for the same reason, CS is most employed in the pharmaceutical field as a coating for drug delivery systems (DDSs) endowed with poor mucoadhesion, which overcomes this limitation through the formation of CS coating [6].

Within the pharmaceutical field, another notable CS application is in brain delivery, where, as is well-known, it is necessary to overcome the formidable obstacle of the blood–brain barrier (BBB), which protects the brain by preventing the entry of toxins, bacteria, and other harmful substances. On the other hand, however, the BBB also limits the entry of potentially useful hydrophilic drugs into the brain since they are unable to overcome such a barrier [7]. Due to CS ability to enlarge cellular tight junctions, it facilitates the paracellular transport of hydrophilic substances, becoming a useful enhancer of their penetration [8]. Hence, CS can be useful for enabling the crossing of biological barriers, such as the BBB, by hydrophilic active principles that should otherwise be ruled out.

Based on the above, CS is an outstanding material for the preparation of DDS, either on its own or combined with other materials.

Currently, CS is obtained by chemical or enzymatic deacetylation of chitin (CH), which, in turn, is available from marine sources. Indeed, crustacean shells of crabs, shrimp, lobsters, and other crustacean wastes are the main sources of CH [9]. However, CS production from marine sources has some limitations due to the fact that the growth of crabs, shrimp, lobsters, and other crustaceans is seasonal and dependent on geographical location [9]. Moreover, for biomedical or food applications of CS from crustaceans, it should be taken into account that CH may interact with micro- and nanoplastic pollution in aquatic ecosystems [10], and may also possess high levels of heavy metals, endotoxins, and proteins [11]. Of course, all these aspects could have important consequences from a toxicological point of view.

Therefore, in the past few years, attention has been focused on an alternative terrestrial source of CH, namely insects, since CH is commonly found not only in the exoskeletons of crustaceans but also in those of other arthropods, such as insects [12]. In particular, the production of CH from insects offers ecological and economic sustainability, with the advantage that insect growth is not seasonal [9]. In addition, insects are highly reproductive and require minimal resources for their growth, providing year-round availability of CH and emerging as a promising, sustainable alternative source of CS [13,14]. Among them, bioconverter insects are able to produce valuable molecules through the bioconversion processes of organic waste substrates, providing sources for CH, protein, and lipid extraction [15,16,17]. One of the most prominent examples of an insect is *Hermetia illucens*, the Black Soldier Fly (BSF), which is the leading farmed insect species employed in waste valorization and feed protein production [18,19].

Recently, we reported on the production and physicochemical and biological characterizations of CS derived from CH produced from *H. illucens* biomasses, which can be deacetylated under heterogeneous and homogeneous conditions. The CS obtained using the two different deacetylation methods differed in the degree of deacetylation (i.e., D.D.) and the distribution of the free amino groups [20,21]. Furthermore, we have also shown that the CS content from these insect sources can vary depending on their developmental stage (i.e., larvae, pupal exuviae, and adults) [21].

As part of our research program aimed at exploring the potential application of CS from insects in the drug delivery, cosmetic, and food fields, in the present work, we validate the previous physicochemical characterization of CS from *H. illucens* larvae deacetylated under heterogeneous conditions. To the best of our knowledge, this study reports, for the first time, the preparation of CS NPs derived from insect sources, specifically from *H. illucens* larvae, for the encapsulation and controlled release of quercetin (QUE).

QUE (2-(3,4-dihydroxyphenyl)-3,5,7-trihydroxychromen-4-one) is a dietary flavonoid with good antioxidant and anti-inflammatory properties [22]. It has gained significant attention in the pharmaceutical, cosmetic, and food industries due to its widely beneficial therapeutic properties [23]. QUE-CS NPs were also synthesized using commercially available CS sourced from crustaceans, enabling a direct comparison of their features.

For that purpose, we determined the average molecular weight (MW) of CS_larvae_ and its crystallinity index (CrI), as well as the crystallite sizes by X-ray diffraction. Furthermore, we have also performed the preparation and physicochemical characterization of quercetin (QUE)-loaded CS_larvae_ nanoparticles (QUE-CS_larvae_ NPs), and evaluated their antioxidant and antimicrobial properties compared with those of the corresponding crustacean source (QUE-CS_crustac_ NPs), employing in both cases CS samples of comparable MW and D.D. Insect-derived chitosan shows comparable behavior compared with commercial crustacean chitosan and, in some aspects, such as nanoparticle size and formulation stability, superior performance. In addition to the environmental and economic benefits associated with using insects as a starting biomass, the results herein support the potential of insect-derived chitosan as an alternative biomaterial for use in nanomedicine and controlled drug delivery systems.

## 2. Materials and Methods

### 2.1. Sample Collection and Materials

*Hermetia illucens* larvae were supplied by Xflies s.r.l. (Potenza, Italy) and reared on a standard Gainesville diet, consisting of 30% alfalfa, 50% wheat bran, and 20% corn meal [24]. Larvae were collected, dried, and ground into powder [25]. Low-molecular-weight commercial chitosan hydrochloride from crustaceans (CS_crustac_) was purchased from Heppe Medical Chitosan GmbH [Halle (Saale), Germany] and, according to the manufacturer’s instructions, had a molecular weight in the range of 60–80 kDa and a D.D. of 90%. Sulfobutylether-β-cyclodextrin sodium salt (SBE-β-CD, MW 2163 Da, and average substitution degree 6.40) was kindly donated by CyDex Pharmaceuticals, Inc. (Kansas, MO, USA). Quercetin free base (QUE), Tween^®^ 85, as well as the salts used for buffer preparation, were purchased from Sigma-Aldrich (Milan, Italy). Throughout this work, double-distilled water was used. All other chemicals used were of reagent grade.

### 2.2. Chitin Extraction

CH extraction from *H. illucens* larvae was carried out based on the process previously reported [25,26].

In order to remove minerals, mainly calcium carbonate, the biomass was treated with formic acid (Sigma-Aldrich, St. Louis, MO, USA). The demineralized material was then filtered, washed to a neutral pH, and dried. Proteins were removed by processing the sample with 2 M sodium hydroxide (NaOH) (Sigma-Aldrich, St. Louis, MO, USA). This phase resulted in unbleached CH, which was then treated with hydrogen peroxide at 90 °C. After filtration, washing, and further drying, bleached CH from *H. illucens* larvae was obtained.

### 2.3. Chitosan Production

CS (CS_larvae_) was obtained through the heterogeneous deacetylation of bleached CH extracted from *H. illucens* larvae. The CH sample was suspended in a concentrated NaOH solution and processed following the method already described [25]. The deacetylated material was poured into acetic acid (CH_3_COOH) for solubilization and centrifuged. The supernatant was collected, and the resulting solution was adjusted to pH 8 to make chitosan insoluble and to be able to recover it. The mixture was incubated overnight at 4 °C to facilitate the biopolymer precipitation. Finally, the precipitated CS was collected through a second centrifugation, washed, freeze-dried, and stored at room temperature. The resulting CS D.D. was determined according to the potentiometric method described in Triunfo et al. [25], and it was equal to 87%.

### 2.4. Chitosan Yield and Molecular Weight

As previously reported in [21,25], CS_larvae_ yield was calculated according to Equation (1):

(1) $$Chitosan\;yield\;\left(\%\right)=\frac{Chitosan\;weight\;after\;freeze-drying\;\left(g\right)}{Dry\;weight\;of\;the\;original\;insect\;sample\;\left(g\right)}*100\;$$

The viscosity-average molecular weight (Mv) of CS_larvae_ was calculated by measuring the intrinsic viscosity (η) of the CS solution using a Ubbelohde capillary viscometer (Fisher Scientific, Waltham, MA, USA). The Mv was calculated using the Mark–Houwink–Sakurada Equation (2), which correlates intrinsic viscosity and molecular weight [27]:

(2) $$\left[\eta \right]=KMv^{\alpha }$$

where [η] is the intrinsic viscosity, and *K* and α are constants [28]. In detail, *K* and *α* are equal to 0.000096 and 0.877, respectively, for both CS_larvae_ and CS_crustac_ [28,29].

### 2.5. Solid State Studies

CS samples, both from *H. illucens* larvae and crustacean sources, were analyzed by X-ray diffraction (XRD) analysis and Fourier-transform infrared spectroscopy (FT-IR) in order to characterize them and validate BSF chitosan identity. The X-ray diffraction spectra of the CS samples were recorded with an X-ray diffractometer (X’Pert PRO, Philips, Thermo Scientific, Milan, Italy) with Cu Kα radiation (40 kV, 32 mA). The scans were performed over a 2θ range of 5° to 50°, at a scan speed of 0.04° s^−1^.

Chitosan crystallinity index (*CrI*) was calculated according to Equation (3):

(3) $$CrI\;\left(\%\right)=\frac{Ic-Ia}{Ic}*100$$

where *Ic* denotes the intensity of the most prominent diffraction peak, corresponding to the crystalline region, and *Ia* refers to the lowest intensity between the main peaks, representing the amorphous region.

Crystallite size was determined using the Scherrer Equation (4) [30]:

(4) $$D\;\left(nm\right)=\frac{k\lambda }{\beta cos\Theta }$$

where *D* is the crystallite size (nm), *k* = 0.9, *λ* is the wavelength, *β* is the width at half height of the peak analyzed, and θ is the diffraction angle.

The FT-IR transmission spectra were recorded using a Jasco 460Plus IR spectrometer (Jasco Europe, Cremella, Italy). Scans were performed at a resolution of 4 cm^−1^ with 100 accumulations, and the transmittance (T%) was measured over the wavelength range of 4000–400 cm^−1^. The obtained spectra were analyzed using JASCO Spectra Manager software. CS samples were then mixed with KBr, and the mixture was pressed into tablets with a 1 cm diameter.

In particular, lyophilization over 72 h (Lio Pascal 5P, Milan, Italy) was carried out before acquiring FT-IR spectra of the investigated nanoparticles in the presence (or absence) of QUE. For all tested samples, spectra were obtained in KBr discs using a Perkin Elmer 1600 FT-IR spectrometer (Perkin Elmer, Milan, Italy). The range examined was 4000–1000 cm^−1^, with a resolution of 1 cm^−1^. The control FT-IR spectrum of the QUE powder was also recorded under the same experimental conditions.

### 2.6. Preparation of Quercetin-Loaded Chitosan Nanoparticles (QUE-CS NPs)

All the manipulations involving QUE were carried out under light protection by using vessels covered with aluminum foils. Preliminarily, QUE and SBE-β-CD were allowed to equilibrate in water under stirring at room temperature for 24 h, with a QUE:SBE-β-CD molar ratio of 1:1. In detail, 4.5 mg of SBE-β-CD and 0.63 mg of QUE were co-incubated in 1 mL of double-distilled water at room temperature. Separately, a 0.2% *w*/*v* solution of CS_larvae_ (or CS_crustac_) in aqueous AcOH (1% *v*/*v*) was prepared, and the resulting mixture was maintained under magnetic stirring for a few minutes. Then, according to the ionic gelation method [31,32], 0.2 mL of the filtered QUE/SBE-β-CD mixture was added to 1.5 mL of the polycation solution, as required, to induce the gelation process [33].

All the NPs were isolated by centrifugation (16,000× *g*, 45 min, Eppendorf 5415D, Germany), resuspended in ultrapure water by manual shaking, and characterized for size, zeta potential, encapsulation efficiency percentage (E.E.%), antioxidant release, and the pH of the relative nanosuspensions. In particular, pH values were measured at 25 °C by a FIVEEASY pH meter (Mettler-Toledo, Polaris Parkway, Columbus, OH 43240, USA).

The physical mixture of QUE/SBE-β-CD was prepared by weighing equimolar amounts of each compound and by mixing the corresponding pulverized powders in a mortar without any solvent.

### 2.7. Quantitative Determination of QUE

The quantitative determination of QUE in each type of CS NP was carried out by spectrophotometric analysis [22,34], and a calibration curve obtained by dissolving QUE in ethanol (concentration range 11–0.11 mg/mL, R^2^ > 0.999) was used. The measurements were performed at a wavelength of 375 nm using a Shimadzu 1900i UV-VIS spectrophotometer (Shimadzu, Milan, Italy). The QUE E.E.% was calculated by Equation (5):

(5) $$E.E.\;\left(\%\right)=\frac{Total\;QUE-QUE\;in\;the\;supernatant\;after\;centrifugation}{Total\;QUE}*100$$

where total QUE is intended as the starting amount of the antioxidant used for NP preparation, and QUE refers to the antioxidant in the supernatant after centrifugation. The study was performed in triplicate.

### 2.8. Physicochemical Characterization of the QUE-CS NPs

Particle size and polydispersity index (PDI) of all investigated NPs were measured at 25 °C after dilution in double-distilled water (1:1, *v*:*v*) using a Zetasizer NanoZS (ZEN 3600, Malvern, UK) apparatus in photon correlation spectroscopy (PCS) mode. The zeta potential was also determined at 25 °C using laser Doppler anemometry (Zetasizer NanoZS, ZEN 3600, Malvern, UK) after a dilution of 1:20 (*v*:*v*) in the presence of KCl (1 mM, pH 7). The particle size, PDI, and zeta potential values were each measured in triplicate, and the results are shown as the mean ± SD.

Transmission electron microscopy (TEM, FEI Tecnai 12 TEM, Eindhoven, The Netherlands) was employed to characterize the morphology of the obtained NPs (120 kV, LaB6 filament) and was calibrated with an Agar Scientific S106 cross grating. Particle suspensions were prepared in Milli-Q water and sonicated for 30 min. A 2 μL aliquot of each suspension was then drop-cast onto Formvar-coated Cu grids (300 mesh, Agar Scientific, Rotherham, UK) and air-dried.

### 2.9. Physical Stability of the QUE-CS NPs

QUE-CS_larvae_ NPs and QUE-CS_crustac_ NP water suspensions were maintained and exposed at 4 °C (fridge), and storage was prolonged for three months by adapting, with slight modifications, a protocol already used by us [33,35]. At several times, the NP size and zeta potential were measured as described in Section 2.8. Such experiments were performed in triplicate for each type of CS NP.

### 2.10. In Vitro Release Studies of Quercetin from QUE-CS NPs

The in vitro antioxidant activity of QUE-CS_larvae_ NPs and QUE-CS_crustac_ NPs, as well as the control samples—free QUE, plain CS_larvae_ NPs, and plain CS_crustac_—was evaluated according to a recently published protocol [36]. Briefly, freshly prepared QUE-loaded NPs (from CS_larvae_ or CS_crustaceans_), corresponding to 70–200 µg of QUE, were suspended in 1.5 mL of double-distilled water in a dialysis bag soaked in 30 mL PBS (0.01 M, pH 7.4) and thermostated at 37° ± 0.1 °C in an agitated (40 rpm/min) water bath (JULABO, Milan, Italy). At appropriate time intervals, withdrawals of 1.5 mL were carried out and centrifuged at 16,000× *g*, 45 min (Eppendorf 5415D, Germany). Moreover, 1.5 mL of freshly prepared PBS was adopted for replacement. The supernatant was analyzed for the QUE content according to the spectrophotometric protocol reported above. The experiment for each type of NP was performed in triplicate, and results are expressed as means ± standard deviation.

### 2.11. DPPH Test

The in vitro antioxidant activity of QUE-loaded NPs (i.e., based on CS from larvae or crustacean) as well as of the control samples—free QUE, plain NPs (i.e., based on CS from larvae or crustacean), and QUE/SBE-β-CD inclusion complex—was evaluated by using the DPPH test with slight modifications [37,38,39]. Firstly, a solution of DPPH in ethanol was prepared at a concentration of 0.001% (*w*/*v*) and then diluted to 8 × 10^−4^% (*w*/*v*). After freeze-drying the NPs, 0.5 mL of each sample previously redispersed in ethanol was reacted with 2.5 mL of the diluted DPPH solution for 60 min at room temperature in the dark; for each sample, the corresponding absorbance was recorded at a wavelength of 514 nm via the use of a Perkin-Elmer Lambda Bio 20 spectrophotometer (Milan, Italy). Blanks were obtained by filling the cuvettes with 0.5 mL of ethanol and 2.5 mL of the 8 × 10^−4^% (*w*/*v*) solution in DPPH. For all tested formulations, antioxidant activity (AA) was calculated using Equation (6) and expressed in percentages:

(6) $$AA\;\left(\%\right)=\left(\frac{1-As}{Ab}\right)*100$$

where *As* is the sample absorbance and *Ab* is the absorbance of the radical compound

### 2.12. Evaluation of Chitosan Nanoparticles’ Antimicrobial Activity

To further characterize the biological properties of QUE-CS_larvae_ NPs, their antimicrobial activity was evaluated through experiments that were conducted using both CS_larvae_ and CS_crustac_ NPs, with and without QUE, to evaluate the antimicrobial effect of QUE and compare the influence of different sources of CS.

#### 2.12.1. Bacterial Strains and Culture Preparation

The experiments were performed against two bacteria used as Gram-negative and Gram-positive strain models, *Escherichia coli* and *Micrococcus flavus*, respectively. Bacteria were inoculated into Luria Bertani (LB) culture medium, following the protocol reported. In order to perform the antimicrobial assays, bacterial cultures were employed at a concentration of 10^6^ CFUs/mL for both species.

#### 2.12.2. Agar Diffusion Test

An agar diffusion test was conducted to evaluate NP antimicrobial activity on the stock samples. Precisely, QUE-CS NPs were selected at a flavonoid concentration of 2 µg/mL. Bacteria were plated on solidified LB-agar medium; after that, the samples were spotted onto the LB agar plates and incubated at 37 °C for 24 h. The antimicrobial activity was evaluated by measuring the inhibition zone diameter (mm). Sterile water, used as a solvent for NP suspensions, was used as the negative control [21,40].

#### 2.12.3. Evaluation of the Minimum Inhibitory Concentration (MIC) by Microdilution Assay

A microdilution assay was carried out on both *E. coli* and *M. flavus* in order to identify the sample minimum inhibitory concentration (MIC), which is the lowest dose needed to control and stall bacterial growth. Experiments were conducted following the protocol reported in our previous work, with some modifications [40].

For both QUE-CS NPs, the tested flavonoid concentrations ranged from 1 µg/mL to 0.0625 µg/mL. Accordingly, five serial dilutions of each sample were made and tested.

The effect of the NPs on the bacterial growth was assessed by evaluating absorbance values, measured by the spectrophotometer (Bellusco, Italy) at a wavelength of 600 nm.

### 2.13. Statistics

Statistical analyses were carried out by Prism v. 5.0 (GraphPad Software Inc., La Jolla, CA, USA). Data were expressed as mean ± SD. Multiple comparisons were based on one-way or two-way analysis of variance (ANOVA). Either Bonferroni’s or Tukey’s *post hoc* test was carried out.

## 3. Results

### 3.1. Chitin Extraction and Chitosan Production

CH extraction from *H. illucens* larvae, according to the protocol reported in Section 2.2, enabled obtaining bleached CS_larvae_ through a heterogeneous process described in Section 2.3, fulfilling the European Pharmacopeia (XI Ed.) requirement on the appearance of CS for pharmaceutical use, which should be a white or almost white fine powder. Table 1 reports the CS_larvae_ yields related to both bleached CH and raw insect biomass.

Furthermore, regarding molecular weight, CS_larvae_ from *H. illucens* was found to have a low viscosimetric molecular weight of around 61 kDa, corresponding to a viscosity degree of 1.5 dL/g.

### 3.2. Chitosan Characterization

**Figure 1:**
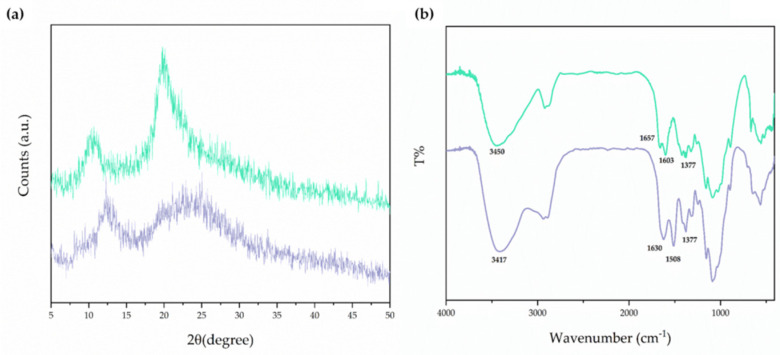
Panel (**a**): XRD patterns of the CS_larvae_ sample extracted from *H. illucens* larvae (green line) and CS_crustac_ (purple line). Panel (**b**): FT-IR spectra of the CSl_arvae_ sample extracted from *H. illucens* larvae (green line) and CS_crustac_ (purple line).

After isolating the polysaccharide matrix CS from *H. illucens* as described in Section 2.3, CS_larvae_ was characterized from a physicochemical point of view by recording its XRD pattern, which was compared with that of CS_crustac_ (Figure 1a).

The two main sharp peaks in the XRD spectra, which are around 10° and 20°, were observed in the case of CS_larvae_ (Figure 1a, green line). Nevertheless, XRD peaks for CS_crustac_ were slightly shifted at higher degrees of diffraction compared with CS_crustac_, but showed lower intensity (Figure 1a, purple line). The characteristic peaks detected were close to those already reported in the literature for CS, both from insects and crustaceans [40,41,42,43,44,45]. XRD analysis also resulted in the assessment of the Cr values of CS_larvae_ and CS_crustac_. The degree of crystallinity was found to be higher for CS_larvae_ than for CS_crustac_ (63% and 42%, respectively, Table 2).

However, the crystallite size was very low for both samples, being in the order of a few nanometers. In both cases, from CrI values, amorphism of CS was derived; in particular, CS_crustac_ seemed more amorphous than CS_larvae,_ maybe due to a more homogeneous distribution of the free amino groups resulting from the deacetylation process.

FT-IR spectrum analysis of bleached CS from *H. illucens* larvae is shown in Figure 1b, together with the FT-IR spectrum of CS_crustac_. This last spectrum of commercial CS was also given in order to make a comparative evaluation of the samples and, at the same time, to assess the identity of the insect biopolymer.

As can be deduced from the FT-IR spectra in Figure 1b, the characteristic bands at 1657 cm^−1^ and 1630 cm^−1^ were both attributed to NH-bending units of glucosamine in the cases of CS_larvae_ and CS_crustac_, respectively (Figure 1b). Furthermore, the N-H and O-H stretching bands in the 3000–3600 cm^−1^ region of the FT-IR spectrum were also in agreement with the previous identification of CS from insects [43]. Overall, FT-IR and XRD spectra confirmed the identity of CS from *H. illucens* and its similarity with the crustacean one.

### 3.3. Preparation and Physicochemical Characterization of the QUE-Loaded CS NPs

Once the characterization of CSl_arvae_ was completed, we dealt with the preparation and physicochemical characterization of QUE-CS_larvae_ NPs, and we compared them to the corresponding QUE-CS_crustac_ NPs, employing in both cases CS samples of comparable low MW and D.D. in order to gain information on the potential application of the former particles in drug delivery. Concerning the preparation of QUE-CS NPs, we used the ionic gelation technique, endowed with several advantages, including ease, low cost, and absence of toxicity [22]. Moreover, in the case of QUE-CS NPs, it has been shown that using high-MW cross-linking agents significantly improved QUE nanoencapsulation efficiency [22]. However, relevant drawbacks that limit the QUE application in pharmaceutical, cosmetic, and food fields are its poor solubility, low bioavailability, and lipophilicity [46,47]. Considering the above, we believe it is of interest to evaluate the effect of an anionic oligosaccharide, such as SBE-β-CD, as a cross-linking agent in the ionic gelation of CS in order to maximize QUE nanoencapsulation efficiency in the polycation. In fact, the positive effect of cyclodextrins (CDs) in improving the solubility and bioavailability of lipophilic substances is well documented in the literature [48].

**Figure 2:**
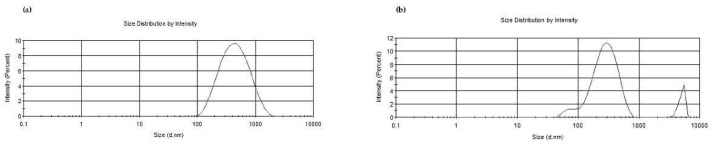
Panel (**a**). Particle size distribution of QUE-CS _larvae_ NPs. Panel (**b**). Particle size distribution of QUE-CS_crustac_ NPs (**b**).

In Table 3, the physicochemical properties of all the prepared NPs are reported. The particle size of CS_larvae_ NPs was not significantly different with or without QUE, whereas plain CS_crustac_ NPs were significantly bigger than the corresponding QUE-loaded NPs (*p* ≤ 0.001, Table 3). Moreover, a more homogeneous particle distribution was noted for the NPs prepared from CS_larvae_ than those from CS_crustac_, as can be seen from the size distributions plotted in Figure 2. Again, the PDI values for plain CS_larvae_ NPs and plain CS_crustac_ NPs were found to be quite different, being equal to 0.25 ± 0.02 and 0.59 ± 0.08, respectively, whereas, in the presence of QUE, the PDI values were very similar (Table 3). For all tested particles, the zeta potential values were positive, ranging from +7.5 mV to +39.3 mV, suggesting a variable external localization of the polycation CS. In particular, a reduction in the positive surface charges of CS was noticed when QUE-loaded NPs were prepared, especially for QUE-CS_crustac_ NPs, which showed a positive surface charge of +7.5 ± 1.1 mV, and started from +27.0 ± 0.5 mV for plain CS_crustac_ NPs. These zeta potential results suggest a greater physical stability of the QUE-CS_larvae_ NPs than the corresponding QUE-CS_crustac_ NPs. As for the E.E.% of QUE, Table 3 shows that the antioxidant agent is nanoencapsulated in a slightly higher amount in QUE-CS_larvae_ NPs than in the counterpart from the crustacean source, even though the differences are not statistically significant (*p* > 0.05 by one-way ANOVA and Tukey’s multiple comparison *post hoc* test). In fact, the QUE E.E.% values observed were in the range of 77 ± 7% and 65 ± 3% for QUE-CS_larvae_ NPs and QUE-CS_crustac_ NPs, respectively. Moreover, the pH of each nanosuspension was assessed and, as shown in Table 3, the pH values measured ranged from 2.7 to 3.3, as expected, considering that CS (larvae or crustacean) was previously dissolved in acidic medium.

### 3.4. Nanoparticle Morphology

**Figure 3:**
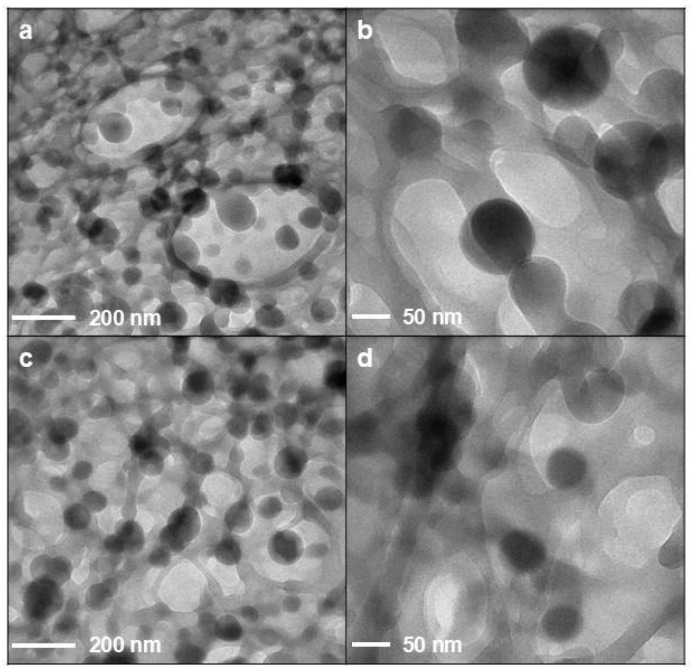
TEM images of NP formulations: QUE-CS_larvae_ NPs (**a**,**b**); QUE-CS_crustac_ NPs (**c**,**d**).

Figure 3 deals with selected TEM micrographs for QUE-CS NPs. No appreciable differences between the two samples were detected by TEM. Regardless of the presence of CS_larvae_ NPs (Figure 3a,b) or CS_crustac_ NPs (Figure 3c,d), it is possible to observe spherical particles with an approximate size of 100–150 nm. In both samples, particles are surrounded by a network of organic matter that could be ascribed to the presence of weakly associated or loosely entangled CS polymer chains.

### 3.5. FT-IR Analysis

**Figure 4:**
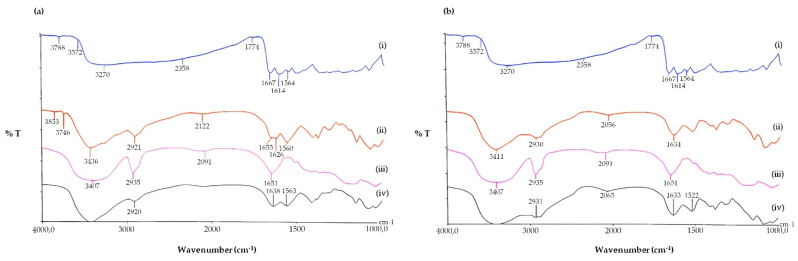
FT-IR spectra of the tested QUE and NP formulations. (**a**) Pure QUE (i); plain CSl_arvae_ NPs (ii); QUE/SBE-β-CD inclusion complex (iii); QUE-CS_larvae_ NPs (iv); (**b**) pure QUE (i); plain CS_crustac_ NPs (ii); QUE/SBE-β-CD inclusion complex (iii); QUE-CS_crustac_ NPs (iv).

FT-IR spectroscopy was used primarily to gain information on the possible inclusion complex formation of QUE with SBE-β-CD. For this purpose, we recorded the FT-IR spectrum of the lyophilized sample resulting from 24 h incubation of equimolar amounts of QUE and SBE-β-CD in double-distilled water (Section 2.6) (Figure 4); we found that it corresponded to that reported in [49] for the QUE/SBE-β-CD inclusion complex. On the other hand, the FT-IR of the physical mixture of QUE/SBE-β-CD (Figure S1) was different enough from that reported in [49] for the QUE/SBE-β-CD inclusion complex. When the physical mixture of SBE-β-CD and QUE underwent FT-IR spectroscopy (Figure S1), the crystalline order of QUE was clearly noticed in the sample, in contrast to the amorphism evidenced in the spectrum of the complex (Figure 4a, iii). In the physical mixture, in fact, a more crystalline order was deduced from the shape of the bands at 1666, 1606 cm^−1^, referring to the C=O stretching vibration and the C=C double bond in the benzene ring of the QUE (Figure S1). Hence, we concluded that the mentioned lyophilized sample consists of the inclusion complex as described elsewhere [49].

Moreover, FT-IR spectroscopy was also used to confirm the occurrence of QUE nanoencapsulation in CS, and the corresponding results are shown in Figure 4. In this Figure, the FT-IR spectra of the mentioned particles are reported together with the absorption spectra of pure QUE and its inclusion complex in SBE-β-CD. When the FT-IR spectrum of free QUE was examined, it displayed a broad absorption band at 3270 cm^−1^, attributable to the phenolic hydroxyl groups of the QUE, a peak at 1667 cm^−1^ corresponding to the C=O stretching vibration, and a peak at 1614 cm^−1^, which represents the C=C double bond in the benzene ring of the QUE [49]. In the FT-IR spectra of both plain NPs, the external localization of the CS chains was deduced from the presence of bands at 1655 cm^−1^ and 1634 cm^−1^, attributable to the NH-bending unit of glucosamine, for CSl_arvae_ and CS_crustac_, respectively (Figure 4a,b, curves ii). Interestingly, for QUE-loaded NPs, the disappearances of the QUE bands at 1667 cm^−1^ and 3270 cm^−1^, together with the retention of the CS bands due to the NH-bending unit of glucosamine at 1638 cm^−1^ and 1633 cm^−1^ (Figure 4a,b, curves iv), constitute evidence of the occurrence of nanoencapsulation [50].

### 3.6. Physical Stability of NPs

**Figure 5:**
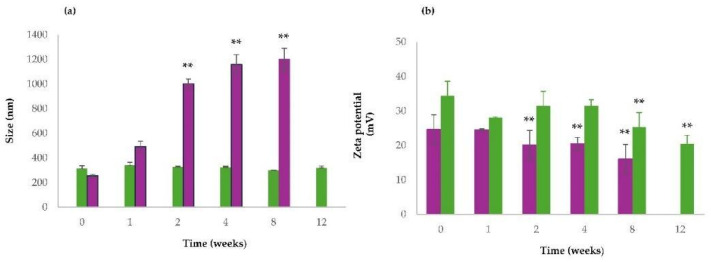
Size stability studies of QUE-CS_larvae_ NPs and QUE-CS_crustac_ NPs (**a**) and zeta potential values changes over time of QUE-CS_larvae_ NPs and QUE-CS_crustac_ NPs (**b**) at 4 °C. QUE-CSl_arvae_ NPs (green bars); QUE-CS_crustac_ NPs (purple bars). Controls were the mean particle size of QUE-CS_larvae_ NPs and QUE-CS_crustac_ NPs, as reported in Table 3. ** *p* ≤ 0.001 as compared with size and zeta values of QUE-CS_larvae_-NPs and QUE-CS_crustac_ NPs reported in Table 3.

An assessment of the physical stability of QUE-CS_larvae_ NPs and QUE-CS_crustac_ NPs at 4 °C was performed by measuring their particle size and zeta potential values during storage. The corresponding outcomes are shown in Figure 5. As can be seen, QUE-CS_larvae_ NPs were more stable than QUE-CS_crustac_ NPs as demonstrated by the finding that the mean diameters of the former particles were almost kept constant over 3 months of storage (Figure 5a), while for the latter particles, relevant aggregation phenomena were observed, even after two weeks of storage at 4 °C (Figure 5a). In addition, as shown in Figure 5b, a progressive and significant decrease in zeta potential values during storage at 4 °C was observed. Overall, for all tested NPs, irrespective of CS, no color change took place over the time of storage, and, hence, the hypothesis of the possible QUE decomposition during storage should be ruled out.

### 3.7. In Vitro Release Profile of QUE from CS NPs

**Figure 6:**
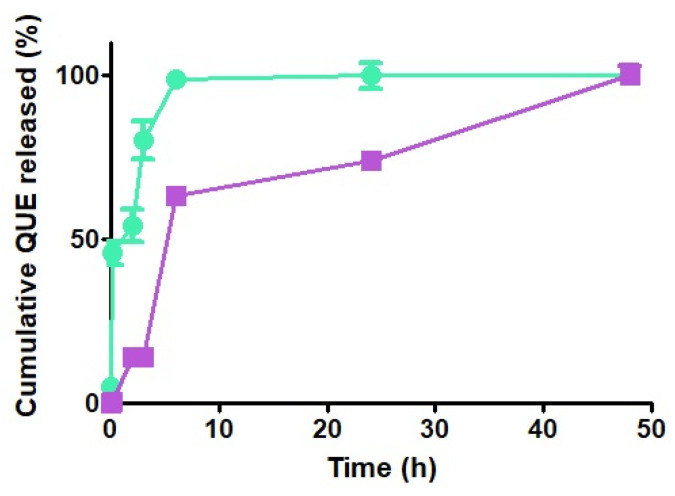
In vitro release profiles of QUE from QUE-CS_larvae_ NPs (green) and QUE-CSc_rustac_ NPs (purple) in PBS at 37 °C.

The in vitro release kinetics of QUE from NPs were determined in PBS (pH 7.4) without enzymes, and relevant differences were observed in the behavior of the two types of CS forming NPs, as shown in Figure 6. While QUE-CS_larvae_ NPs displayed a prompt release of the antioxidant agent (burst effect) reaching the quantitative release within 6 h, the QUE release from QUE-CS_crustac_ NPs was biphasic, being characterized by an initial prompt release reaching 63% of QUE released, followed by a slower release kinetics of the antioxidant, providing the quantitative QUE amount only after 48 h (Figure 6). Even during the course of these experiments for all tested NPs, irrespective of the type of CS, no color change took place, ruling out the hypothesis of the possible QUE decomposition.

### 3.8. DPPH Assay for the Evaluation of Antioxidant Activity

The antioxidant activity of QUE-CS_larvae_ NPs, QUE-CS_crustac_ NPs, and the QUE/SBE-β-CD inclusion complex was studied by exploiting a slightly modified spectrophotometric assay based on the use of the 2,2-diphenyl-1-picrylhydrazyl (DPPH) free radical. In addition to the QUE-loaded DDS in this test, the starting CS powders (i.e., CS_larvae_ and CS_crustac_) and plain CS_larvae_ and CS_crustac_ NPs were included as controls. In this assay, antioxidants are able to reduce the stable DPPH radical (purple) to the nonradical form DPPH (yellow). As reported in Table 4, QUE-CS_larvae_ NPs, QUE-CS_crustac_ NPs, and the QUE/SBE-β-CD inclusion complex showed 100% DPPH radical scavenging activity. The DPPH radical is a stable free radical that generates a deep violet solution in organic solvents. Its progressive reduction in color intensity in the presence of free QUE (and NPs from both CS_larvae_ and CS_crustac_) indicated that QUE was acting as an antioxidant, although nanoencapsulated. Interestingly, however, at the concentrations used, even the starting CS_larvae_ and CS_crustac_ and plain CS_larvae_ NPs and CSc_rustac_ NPs were able to elicit quantitative DPPH radical scavenging activity and, hence, they too were endowed with antioxidant activity (Table 4). Most likely, this last result could be rationalized by considering that CS is characterized by antioxidant activity, as demonstrated by studies showing that CS films can be utilized as active packaging to prevent the oxidation of food products and that the biopolymer CS isolated from marine and terrestrial (mushroom) sources possesses high antioxidant activity [51,52,53].

### 3.9. Biological Evaluation of QUE-Loaded NPs

#### 3.9.1. Agar Diffusion Assay

**Figure 7:**
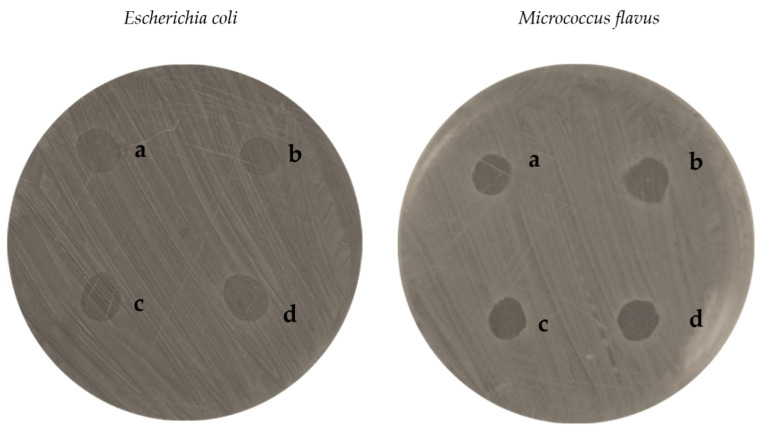
Inhibition zones of QUE-CS_larvae_ NPs (a), plain CS_larvae_ NPs (b), QUE-CS_crustac_ NPs (c), plain CS_crustac_ NPs (d) on *E. coli* and *M. flavus*, resulting from the agar diffusion test.

Results of the agar diffusion assay performed on *E. coli* and *M. flavus* are reported in Table 5. CS_larvae_ NPs as well as CS_crustac_ NPs, both with and without QUE, induced the formation of measurable inhibition zones against both bacterial species tested. Sterile water showed no inhibition zones (Figure 7; Table 5).

**Figure 8:**
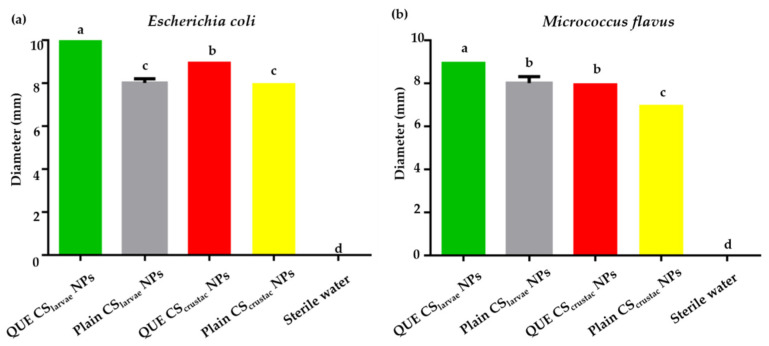
(**a**) Results from agar diffusion test of QUE-CS_larvae_ NPs (green bar), plain CS_larvae_ NPs (gray bar), QUE-CS_crustac_ NPs (red bar), and plain CS_crustac_ NPs (yellow bar) on *E. coli*; (**b**). Results from agar diffusion test of QUE-CS_larvae_ NPs (green bar), plain CS_larvae_ NPs (gray bar), QUE-CS_crustac_ NPs (red bar), and plain CS_crustac_ NPs (yellow bar) on *M. flavus*. Different letters indicate significant differences (*p* < 0.05) among treatments. Data were analyzed with one-way ANOVA and Bonferroni’s *post hoc* test.

On *E. coli*, QUE-CS_larvae_ NPs generated the largest inhibition zone, approximately 10 mm in diameter, which was also found to be larger than that obtained from the sample of QUE-CSc_rustac_ NPs (9 mm, Figure 8a). This result was statistically significant compared with the QUE-CS_crustac_ NPs. Indeed, plain CSl_arvae_ NPs and plain CS_crustac_ NPs showed lower antimicrobial efficacy against the bacterium, with inhibition diameters of approximately 8 mm (Figure 8a).

The same pattern was observed with respect to *M. flavus*, with QUE-CS_larvae_ NPs again resulting in the highest inhibition zone (9 mm, Figure 8b). The differences were still statistically significant. The absence of any antibacterial activity of the negative control (sterile water) and the lower activity detected for plain NPs (both from CS_larvae_ NPs and CS_crustac_ NPs) confirmed that the inhibitory effect was positively enhanced by the presence of the flavonoid QUE and the structure of the NPs themselves, which consist of a biopolymer that possesses antibacterial activity.

#### 3.9.2. Microdilution Assay

After the antibacterial activity in solid assays was assayed by the agar diffusion test, NPs were characterized for their antibacterial activity in the liquid phase using a microdilution assay.

**Figure 9:**
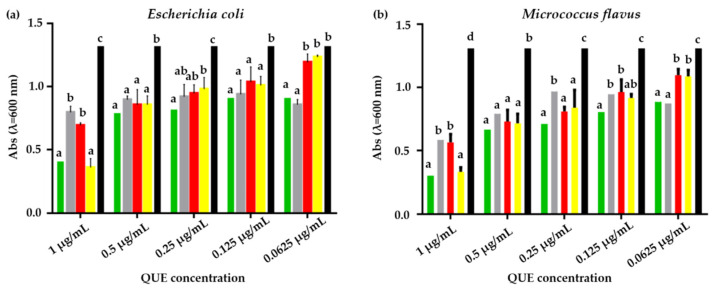
(**a**). Results of microdilution assay for QUE-CS_larvae_ NPs (green bar), plain CS_larvae_ NPs (gray bar), QUE-CS_crustac_ NPs (red bar), plain CS_crustac_ NPs (yellow bar), and the control (black bar) at five concentrations, 1, 0.5, 0.25, 0.125, and 0.06250 µg/mL against *E. coli.* (**b**). Results of microdilution assay for QUE-CSl_arvae_ NPs (green bar), plain CSl_arvae_ NPs (gray bar), QUE-CS_crustac_ NPs (red bar) and plain CS_crustac_ NPs (yellow bar), and the control (black bar) at five concentrations, 1, 0.5, 0.25, 0.125, and 0.06250 µg/mL against *M. flavus*. Data are expressed as mean ± standard deviation. Different letters indicate significant differences (*p* < 0.05) between absorbance values of the bacterial culture alone and that of bacteria treated with different concentrations of each treatment with QUE or without QUE (data were analyzed using two-way Anova and the Bonferroni post hoc test).

Microdilution assay performed on *E. coli* revealed a reduction in microbial growth for all samples of CS_larvae_ NPs and CS_crustac_ NPs, both QUE-loaded and plain (Figure 9a,b). At a 1 µg/mL concentration of QUE, QUE-CS_larvae_ NPs lowered microbial growth in a statistically significant way compared to both plain CS_larvae_ NPs and pure CS_larvae_ (Figure 9a,b).

This statistical significance was also found when compared to QUE-CS_crustac_ NPs, while plain CS_crustac_ NPs reduced *E. coli* growth similarly to the QUE-CS_larvae_ NPs. At the 0.5 µg/mL QUE concentration, all samples showed statistically significant efficacy compared to the negative control (bacterial suspension without treatment), while showing no significant differences compared to each other. By further halving the concentration, all samples were effective compared with the control. Treatment with QUE-CS_larvae_ NPs, although not significantly, was shown to most strongly downregulate bacterial growth. At a QUE concentration of 0.125 µg/mL, no difference was found among the samples, but they were all effective compared to the bacterial culture. At the lowest QUE concentration (0.0625 µg/mL), both plain CS_larvae_ NPs and QUE-CS_larvae_ NPs showed a statistically significant bacterial growth reduction compared to QUE-CS_crustac_ NPs and plain CS_crustac_ NPs (Figure 9a,b). Altogether, the experiments revealed an inhibitory activity of plain CS_larvae_ NPs at concentrations up to 0.062 µg/mL, but it was not possible to find an MIC value that was statistically significant compared to plain CS_crustac_ NPs and QUE-CS_crustac_ NPs. This suggests that the effective MIC value in these terms could be slightly higher than the range studied in this work.

Within the same experiment, conducted on *M. flavus* at the highest QUE concentration (1 µg/mL), a significant inhibition of bacterial growth was found following treatment with all the samples, with a higher inhibition due to the QUE-CS_larvae_ NPs. At a 0.5 µg/mL QUE concentration, all samples gave statistical significance compared to the control, but not compared to each other. By further halving the QUE concentration, QUE-CS_larvae_ NPs provided the highest inhibition and were significantly different from plain CS_larvae_ NPs. With regard to the last and lowest QUE concentrations tested, QUE-CS_larvae_ NPs showed relevant efficacy at 0.125 µg/mL, which was statistically different from plain CS_larvae_ NPs and plain CS_crustac_ NPs (Figure 9b). Concerning the activity of the samples on *M. flavus*, the MIC value for QUE-CS_larvae_ NPs could be detected at a flavonoid concentration of 0.125 µg/mL, which showed a statistically significant difference compared with plain CSl_arvae_ NPs.

## 4. Discussion

The present work was carried out with the following objective: *(i)* to validate the characterization of CS derived from *H. illucens* larvae deacetylated under heterogeneous conditions, and *(ii)* to compare the physicochemical, antioxidant, and antimicrobial characteristics of QUE-CSl_arvae_ NPs with those corresponding to QUE-CS_crustac_ NPs arising from the crustacean source, employing in both cases CS samples of low MW and comparable D.D. This last aim should be set in the context of exploring the application potential of CS from insects in the drug delivery, cosmetic, and food fields. Herein, the optimization of the extraction processes—likely linked to better temperature regulation that mitigated polymer degradation during the deacetylation phase—resulted in a slightly higher MW value than that identified and reported in Triunfo et al., [25] for the same CS from *H. illucens* larvae.

MW can greatly affect the physicochemical properties and biological activity of CS [54], and low-MW CS, often related to a high D.D., exhibits higher antimicrobial and anti-inflammatory capacities [55] as well as great scavenging free radical activity [56]. Moreover, as reported in Table 1, the CS yield obtained in this work is around 38% from the respective CH and 3% from the initial biomass. Under similar reaction conditions (reaction time, temperature, and alkali concentration), the yield from CH in this study is slightly higher than the 33% reported in Triunfo et al., [25], likely due to the optimization of the extraction processes and improved biopolymer purification. However, the final yield obtained from the starting biomass remains around 3%, consistent with that reported in Triunfo et al., [25]. This value also aligns with what has been reported in the literature for insect CS [25] and is comparable (albeit slightly lower) to the yields reported in the literature for CS from crustaceans [57,58,59]. Insects have a higher lipid and protein content compared to crustaceans [25,60], which reduces the final biopolymer yield from the starting biomass. Overall, the biopolymer yield, primarily determined by the initial CH yield, also depends on the insect species, developmental stage, and body parts used [61,62].

To complete the characterization of CS derived from *H. illucens* larvae, the most noteworthy result obtained herein is the greater crystallinity of CS_larvae_ compared with CS_crustac,_ with CrI (%) values of 63% and 42% for the former and the latter, respectively (Table 2). The crystallinity index of a polymer matrix in the context of drug delivery is believed to affect important features, such as its solubility, dissolution rate, and physical stability of the polymer matrix. In general, more crystalline polymers exhibit greater physical stability, while higher solubility and drug release rates have been observed in polymers with a low degree of crystallinity.

Concerning the QUE-loaded CS NPs, QUE-CS_larvae_ NPs showed distinct features compared with QUE-CS_crustac_ NPs. In fact, it was noted that, while the particle size of CS_larvae_ NPs was not significantly different in the presence or absence of QUE (either free or as a QUE/SBE-β-CD inclusion complex), a significant decrease in particle size was observed for QUE-CS_crustac_ NPs compared with the corresponding plain CS_crustac_ NPs. At this stage, it is not simple to explain this outcome, but our hypothesis, to be fully demonstrated, is that the QUE presence (either free or as QUE/SBE-β-CD inclusion complex) during the course of the preparative process of QUE-loaded CS_crustac_ NPs by ionic gelation brought about a conformational reorganization of the CS_crustac_, leading to NP shrinkage. In particular, considering that *(i)* the particle size of QUE-loaded CS_crustac_ NPs depends on several factors, including pH, volume CS/volume cross-linker ratio, type of cross-linker, preparative method, and so on; and *(ii)* QUE is a substance that may be involved in several electrostatic interactions with the positively charged amino-groups of CS, it follows that more or less compact structures can be formed as described in the literature [63,64]. Then, taking into account that CS_larvae_ is characterized by a CrI value greater than that of CS_crustac_, the conformational freedom of the former biopolymer chains should be more restricted than that of the latter; hence, from CS_larvae_, less compact structures should arise (i.e., larger particle sizes), while from CS_crustac_, more compact structures should arise (i.e., smaller particle sizes).

Moreover, QUE-CS_larvae_ NPs showed a monodisperse particle size distribution, unlike those from CS_crustac_, as can be seen in Figure 2. This may be advantageous because monodisperse nanoparticles show more homogeneity in size, shape, and surface properties.

As for the zeta potential of QUE-loaded and unloaded particles, a sharp difference was noted between QUE-CSl_arvae_ and CS_crustac_. NP refers to an increase in zeta potential for the former and a decrease in zeta potential for the latter particles with respect to the corresponding plain particles. While QUE-CS_larvae_ NPs showed a highly positive zeta potential (+39.3 ± 1.8 mV), the zeta potential value of QUE-CS_crustac_ NPs was slightly positive (+7.5 ± 1.1 mV). This suggests that QUE-CS_larvae_ NPs are much more physically stable than QUE-CS_crustac_ NPs, as also confirmed in our storage stability studies (Figure 5) [65]. In this regard, our storage stability study has contributed to advancing knowledge in this field, demonstrating that QUE-CS_larvae_ NPs maintain features indicative of stronger colloidal stability even for three months (Figure 5). This represents a step forward compared to other commercial chitosan delivery systems, such as QUE lecithin–CS NPs, which maintained physicochemical integrity for 28 days at 4 °C [66], or chitosan-coated resveratrol–QUE NPs, which retained size and zeta potential over 5 days at 4 °C [67]. In addition, these results also indicate that in QUE-loaded CS_larvae_ NPs, the positively charged amino groups should be localized on the surface of the particles to a higher extent than in QUE-CS_crustac_ NPs. As can be easily deduced from the above discussion, the observed increase in zeta potential for QUE-CS_larvae_ NPs, compared with the decrease for QUE-CS_crustac_ NPs, is crucial for understanding what is noted about nanoparticle stability, and there is a need to fully characterize the localization of positively charged amino groups of CS_larvae_ on the particle surface. More direct evidence in this regard should be obtained in future studies by X-ray photoelectron spectroscopy (XPS), which, as is known, analyzes the surface elemental composition of particles.

Moreover, even though not statistically significant (see Table 3), the greater E.E.% noted for QUE-CS_larvae_ NPs compared with QUE-CS_crustac_ NPs may be related to the greater localization of positively charged amino groups of CS_larvae_ on the surface of the former particles. These increased positive surface charges, indeed, may induce a greater amount of flavonoid QUE encapsulation by electrostatic interactions at the outer shell of QUE-CS_larvae_ NPs compared to the corresponding CS_crustac_-based NPs.

Moreover, it is interesting to discuss the findings of the present work in light of those reported by Kim and coworkers, who investigated the effects of different cross-linkers on the E.E.% of QUE in the preparation of QUE-loaded chitosan nanoparticles (CS_crustac_ NPs) by ionic gelation [24]. In particular, these authors studied the E.E.% of QUE-CS_crustac_ NPs prepared by ionic gelation at pH 3.5 with a low-MW cross-linker (i.e., tripolyphosphate (367.9 Da)) as well as with some macromolecular cross-linkers such as dextran sulfate (>15 kDa), arabic gum (AG, >250 kDa), and hyaluronic acid (HA, >1000 kDa). Interestingly, it has been found that the highest E.E.%—92.5% ± 2.7, 94.8% ± 0.8 to 98.3% ± 0.7—were observed for HA, AG, and dextran sulfate, respectively, while, for the low-MW cross-linker, tripolyphosphate was detected with an E.E.% of 48.7% ± 0.4 only. Hence, cross-linker agents at a high MW positively influence the nanoencapsulation of the flavonoid QUE. As mentioned above, we selected the anionic oligosaccharide SBE-β-CD (MW 2163 Da) as a cross-linking agent to maximize the QUE nanoencapsulation efficiency in CS, considering that CDs improve the solubility and bioavailability of lipophilic substances [49]. As reported in Table 3, under the conditions we used for QUE nanoencapsulation, the E.E.% values of QUE-CS_larvae_ NPs and QUE-CS_crustac_ NPs resulted in 77% ± 7 and 65% ± 3, respectively. These values are slightly lower than those observed with high-MW cross-linkers, but very different from those detected with the low-MW cross-linker tripolyphosphate.

A further aspect considered by us refers to the well-known QUE decomposition since it is susceptible to chemical and enzymic attacks, leading to modification of its biological properties. Moreover, it is also known that this decomposition is significantly influenced by pH, occurring faster in an alkaline medium, while the flavonoid is generally more stable at lower pH values [68]. This pH-dependent degradation can affect the stability of QUE in various applications, such as the development of QUE-loaded DDS and food preservation. As can be seen from Table 3, the pH values of QUE-CS_larvae_ NPs and QUE-CS_crustac_ NP nanosuspensions were acidic (i.e., 3.2 ± 0.03 and 2.7 ± 0.01, respectively), and, hence, the loaded QUE in both formulations is supposed to have significant stability toward pH-dependent degradation. Future studies will be devoted to clarifying the role exerted by the acidic pH medium in terms of QUE levels during storage over time.

Finally, it is noteworthy that the above-mentioned finding—that in QUE-loaded CS_larvae_ NPs, the positively charged amino groups are localized on the surface of the particles, while in QUE-CS_crustac_ NPs, the same positively charged groups should be located in lower amounts out of the particles—is fully in agreement with the observed in vitro release profiles of the antioxidant from CS NPs. Indeed, the prompt release of the antioxidant agent (burst effect) observed for the QUE-CS_larvae_ NPs is consistent with the previously said greater amount of flavonoid QUE encapsulation by electrostatic interactions at the outer shell of QUE-CS_larvae_ NPs (Figure 6). Similarly, the biphasic QUE release from QUE-CS_crustac_ NPs, characterized by an initial prompt release followed by sustained release kinetics of the antioxidant, is consistent with the main QUE encapsulation located in the core of the particles and, in the lower part, at the outer shell of QUE-CS_crustac_ NPs (Figure 6). Hence, we make it clear that a decisive role is played by the positive amino group localization more than the crystallinity index differences (63% vs. 42%) between the two types of CS examined. In the literature, it was found that, from CS_crustac_ NPs containing the SBE-β-CD/QUE inclusion complex, QUE release was only by 40% at neutral pH [69,70], which is far lower than the release percentage obtained with the use of CS derived from *H. illucens* larvae in the present work. However, in these two studies, a similar but less intense initial burst release of QUE was observed, as occurred in our QUE-CS_larvae_ NPs. In this context, CS_larvae_ not only represents a more sustainable product, but is also more suitable for enhancing bioactive release under specific conditions with neutral pH.

From a biological viewpoint, CS from *H. illucens* larvae has already been demonstrated to show antibacterial activity against both Gram-negative and Gram-positive bacteria [40], and, hence, encapsulating QUE in such CS was expected to enhance this crucial biological property.

Results obtained from the agar diffusion test demonstrated a synergic effect that resulted from QUE and the CS matrix employed to produce NPs (Figure 7 and Figure 8a,b). For both *E. coli* and *M. flavus*, the activity of QUE-loaded NPs was slightly higher than the corresponding plain NPs, irrespective of the type of CS. The mutual comparison of antibacterial susceptibility among the plates was slightly stronger on *E. coli* than *M. flavus*, in terms of inhibition zone, with enhanced activity resulting from QUE-CS_larvae_ NPs compared with QUE-CS_crustac_ NPs. Currently, it is not possible to make a direct comparison with similar studies because, to the best of our knowledge, this is the first work in which the antibacterial activity of QUE-CS_larvae_ NPs has been tested. For this reason, it is only possible to compare the data obtained based on the studies carried out on nanosystems with commercial chitosan. Indeed, other research trials have already demonstrated the antibacterial activity of QUE-CS_crustac_ NPs, obtaining slightly higher inhibition zones than those found in this work (12 mm), but at a higher concentration [71]. It can be hypothesized that nanoformulations interact with bacterial cells, promoting the internalization of drug-loaded nanoparticles, which may lead to membrane disruption, enzyme inactivation, and structural alterations, thereby enhancing antibacterial efficacy [72]. Li et al. [73] also investigated the antibacterial activity of chitosan nanoparticles containing QUE and found inhibition zones larger in diameter than those found on both Gram-negative and Gram-positive bacteria, likely also related to the presence of catechol in the nanosystems, a flavanol with proven antibacterial activity. Moreover, QUE-loaded alginate/chitosan nanoparticles were studied in another work [74], and it was found that inhibition zones ranged between 12 and 17 mm on *E. coli*. In this case, the presence of alginate is not a discriminating factor for antimicrobial activity, and the inhibition zones were wider at QUE concentrations that were three times higher than those tested in this work (12 µg/mL vs. 4 µg/mL).

CS produced from *H. illucens* larvae has well-known antibacterial activity against both Gram-negative and Gram-positive bacteria [40]. For the biopolymer from larvae, tested on its own, MIC values were found in the range of 62.5–125 μg/mL on *Staphylococcus epidermidis* (as a Gram-positive model bacterium) and above 500 μg/mL on *E. coli* [74]. Encapsulation of QUE in NPs made of CS_larvae_ enhances this fundamental biological property, further lowering the MIC values compared to the biopolymer tested in the literature.

Indeed, according to the literature, MIC values found for *E. coli* were much higher than the concentrations tested in this study. This supports the thesis that it was not possible to find an MIC that was statistically significant compared with the plain NPs, as the concentrations tested for *E. coli* are probably too low. For instance, Messias de Souza et al. demonstrated that the MIC values of QUE-loaded chitosan nanoparticles evaluated against Gram-negative bacteria yielded inhibition at concentrations between 6.25 and 12.5 mg/mL [75].

Concerning the MIC values, Li et al. found MIC values for the tested Gram-positive bacteria (*Staphylococcus aureus* and *Bacillus subtilis*) in the range of 4.88–9.76 µg/mL, which were higher than the MIC value found for the Gram-positive bacteria in this study, despite the presence of QUE with another bioactive molecule within the nanoparticles [73]. Another study conducted on *S. aureus* also found marked antimicrobial activity of chitosan nanoparticles loaded with QUE and functionalized with hordein, reporting an MIC value higher than that identified in this study (256 ppm) [76].

## 5. Conclusions

To the best of our knowledge, this is the first study that evaluated the comparative effects of CS obtained from *H. illucens* larvae or from a crustacean (commercial) source on the physicochemical characteristics and biological activity of CS based polymeric nanoparticles as DDS. In particular, herein, we investigated QUE-loaded CS NPs using QUE as a lipophilic antioxidant model drug as well as CS from insect and crustacean sources at a low molecular weight (61 kDa and 60–80 kDa, respectively) using SBE-β-CD as a cross-linker agent in the ionic gelation of the polycation CS. The deacetylation degrees of both CS types were very close (87% and 90%, respectively), while the crystallinity indexes of CS_larvae_ and CS_crustac_ were different, being 63% and 42%, respectively. Overall, it was found that the source of CS had a significant impact on the physicochemical properties of the DDS derived from the two biopolymers compared. In particular, the QUE-CS_larvae_ NPs showed a monodisperse particle size distribution, unlike those from CSc_rusta_c. Furthermore, while QUE-CS_larvae_ NPs showed a highly positive zeta potential, QUE-CS_crustac_ NPs were slightly positive, suggesting that QUE-CS_larvae_ NPs are much more physically stable than QUE-CS NPs, as experimentally observed. The increased positive surface charges of QUE-CS_larvae_ NPs may induce a greater amount of flavonoid QUE encapsulation at the outer shell of QUE-CS_larvae_ NPs compared to the corresponding CS_crustac_-based NPs. This differing localization of encapsulated flavonoid QUE (in the outer shell or the core of the particles) is consistent with the observed in vitro release profiles of the antioxidant QUE from CS NPs. As for the DPPH radical scavenging activity, each NP formulation, QUE/SBE-β-CD inclusion complex, or the pure polysaccharides showed essentially quantitative antioxidant activity, most likely due to the inherent antioxidant activity of the CS examined, which hinders their comparison. The studied nanosystems showed great antibacterial properties, likely related to the use of CS as a matrix of the nanosystems. However, this activity was more intense for QUE-CSl_arvae_ NPs, probably due to the synergy between insect chitosan, which has considerable antimicrobial properties, and the encapsulated QUE.

The results demonstrated the notable potential of CS derived from *H. illucens* larvae as a source of low-molecular-weight CS to be employed in the development of DDS with interesting physicochemical characteristics. But, before drawing definitive conclusions, further investigation is necessary to evaluate the scope and limitations of this promising approach, which has relevant applications in the drug delivery, cosmetic, and food fields [77].

## Figures and Tables

**Table 1:** Yields (%) related to bleached CH and raw insect biomass of different CS samples heterogeneously deacetylated from bleached CH ^a^.

Starting Material	Yield of CS_larvae_ (%)
Bleached CH	38 ± 0.8
Raw insect biomass	3 ± 0.1

^a^ Data are expressed as mean ± standard deviation.

**Table 2:** Crystallinity index (CrI) resulted from XRD analysis of CS_larvae_ and CS_crustac_.

CS Sample	CrI (%)
CS_larvae_	63
CS_crustac_	42

**Table 3:** Physicochemical properties of the studied NPs.

Formulation	Size ^a^ (nm)	PDI ^b^	QUE E.E. (%)	Zeta Potential (mV)	pH
QUE-CS_larvae_ NPs	307 ± 24	0.40 ± 0.05	77 ± 7	+39.3 ± 1.8	3.2 ± 0.03
QUE-CS_crustac_ NPs	180 ± 17 **	0.39 ± 0.03	65 ± 3	+7.5 ± 1.1 **	2.7 ± 0.01
Plain CS_larvae_ NPs	259 ± 6	0.25 ± 0.02	-	+35.3 ± 0.2	3.3 ± 0.03
Plain CS_crustac_ NPs	547 ± 60	0.59 ± 0.08	-	+27.0 ± 0.5	2. 8 ± 0.07

^a^ Mean ± standard deviation of at least eight replicates (*n* = 8) are reported. For size and zeta measurements, plain CS_larvae_ NPs and plain CS_crustac_ NPs were taken as controls for statistical evaluation for QUE CS_larvae_ NPs and QUE CS_crustac_ NPs, respectively. ^b^ PDI: polydispersity index. ** *p* ≤ 0.001 as compared with plain NPs.

**Table 4:** In vitro antioxidant activity of different QUE-loaded or unloaded NP formulations, the QUE/SBE-β-CD inclusion complex, and pure polysaccharides evaluated by the DPPH assay ^a^.

NP Formulations/Inclusion Complex/Pure Polysaccharides	Antioxidant Activity
QUE-CS_larvae_ NPs	98.8 ± 1.5
QUE-CS_crustac_ NPs	100 ± 2
QUE/SBE-β-CD inclusion complex	100 ± 3
Plain CS_larvae_ NPs	99.7 ± 0.8
Plain CS_crustac_ NPs	100 ± 2
CS_larvae_	100 ± 3
CS_crustac_	100 ± 3

^a^ Data are expressed as mean ± standard deviation of six replicates.

**Table 5:** Diameters (mm) of the inhibition zones determined by QUE-CS_larvae_ NPs, plain CS_larvae_ NPs, QUE-CS_crustac_ NPs, plain CS_crustac_ NPs on *E. coli* and *M. flavus*. Sterile water was tested as a negative control.

Bacterial Species	Sample	Diameter Inhibition zone (mm)	Bacterial Species	Sample	Diameter Inhibition zone (mm)
*E. coli*	QUE-CS_larvae_ NPs	10 ± 0.1 ^a^	*M. flavus*	QUE-CS_larvae_ NPs	9 ± 0.2 ^a^
	Plain CS_larvae_ NPs	8 ± 0.2 ^c^		Plain CS_larvae_ NPs	8 ± 0.3 ^b^
	QUE-CS_crustac_ NPs	9 ± 0.2 ^b^		QUE-CS_crustac_ NPs	8 ± 0.4 ^b^
	Plain CS_crustac_ NPs	8 ± 0.1 ^c^		Plain CS_crustac_ NPs	7 ± 0.1 ^c^
	Sterile water	- ^d^		Sterile water	- ^d^

Results are expressed as the mean ± standard deviation of diameters measured with the agar diffusion test of three independent biological replicates. Different letters indicate significant differences (*p* < 0.05) among treatments.

## Data Availability

The original contributions presented in this study are included in the article. Further inquiries can be directed to the corresponding author(s).

## Supplementary Materials

The following supporting information can be downloaded at https://www.mdpi.com/article/10.3390/pharmaceutics17081016/s1: Figure S1: FT-IR spectra of Pure SBE-β-CD (a); physical mixture SBE-β-CD/QUE (b).
