# Supplementary material for: Physicochemical and Biological Properties of Quercetin-Loaded Low-Molecular-Weight Chitosan Nanoparticles Derived from Hermetia illucens Larvae and Crustacean Sources: A Comparative Study
Source: Pharmaceutics. 2025 Aug 5;17(8):1016. doi: 10.3390/pharmaceutics17081016 (PMC12389238; doi:10.3390/pharmaceutics17081016)
Supplement: Supplementary file 1 [file pharmaceutics-17-01016-s001.zip › pharmaceutics-3767424-supplementary.pdf]

SUPPLEMENTARY MATERIALS

FOR

Physicochemical and Biological Properties of Quercetin-Loaded Low-Molecular-Weight Chitosan Nanoparticles Derived from *Hermetia illucens* Larvae and Crustacean Sources: A Comparative Study

Anna Guarnieri [1,†], Rosanna Mallamaci [2,†], Giuseppe Trapani [3], Dolores Ianniciello [1], Carmen Scieuzo [1,4], Francesco Iannielli [1], Luigi Capasso [3], Maria Chiara Sportelli [5], Alessandra Barbanente [5], Michela Marsico [1], Angela De Bonis [1], Stefano Castellani [6], Patrizia Falabella [1,4,*] and Adriana Trapani [3,*]

1 Department of Basic and Applied Sciences, University of Basilicata, 85100 Potenza, Italy; anna.guarnieri@unibas.it (A.G.); dolores.ianniciello@unibas.it (D.I.); carmen.scieuzo@unibas.it (C.S.); francesco.iannielli003@unibas.it (F.I.); michela.marsico@unibas.it (M.M.); angela.debonis@unibas.it (A.D.B.)
2 Department of Biosciences, Biotechnologies and Environment, University of Bari "Aldo Moro", 70125 Bari, Italy; rosanna.mallamaci@uniba.it
3 Department of Pharmacy-Drug Sciences, University of Bari "Aldo Moro", 70125 Bari, Italy; giuseppe.trapani@uniba.it (G.T.); l.capasso2@phd.uniba.it (L.C.)
4 Spinoff XFlies S.R.L, University of Basilicata, 85100 Potenza, Italy
5 Chemistry Department, University of Bari "Aldo Moro", 70125 Bari, Italy; maria.sportelli@uniba.it (M.C.S.); alessandra.barbanente@uniba.it (A.B.)
6 Department of Precision and Regenerative Medicine and Ionian Area (DiMePRe-J), University of Bari "Aldo Moro", 70125 Bari, Italy; stefano.castellani@uniba.it
* Correspondence: patrizia.falabella@unibas.it (P.F.); adriana.trapani@uniba.it (A.T.); Tel.: +39-0971-205501 (P.F.); +39-080-5442114 (A.T.)
† These authors contributed equally to this work.

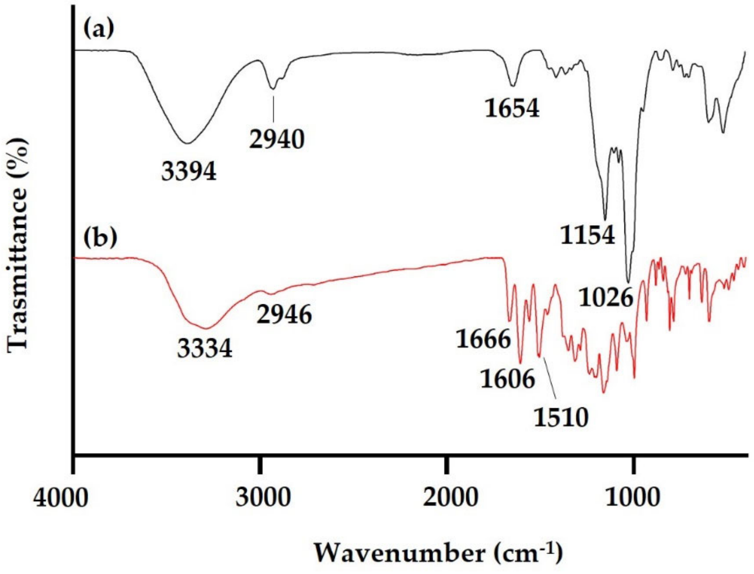

Figure S1. FT-IR spectra of Pure SBE-β-CD (**a**); physical mixture SBE-β-CD/QUE (**b**).
